# Supplementary material for: Longitudinal Plasma Lipidomics Reveals Distinct Signatures Following Surgery in Patients with Glioblastoma
Source: Metabolites. 2025 Oct 15;15(10):673. doi: 10.3390/metabo15100673 (PMC12566518; doi:10.3390/metabo15100673)
Supplement: Supplementary file 1 [file metabolites-15-00673-s001.zip › metabolites-3801777-supplementary.pdf]

Supplementary Figures

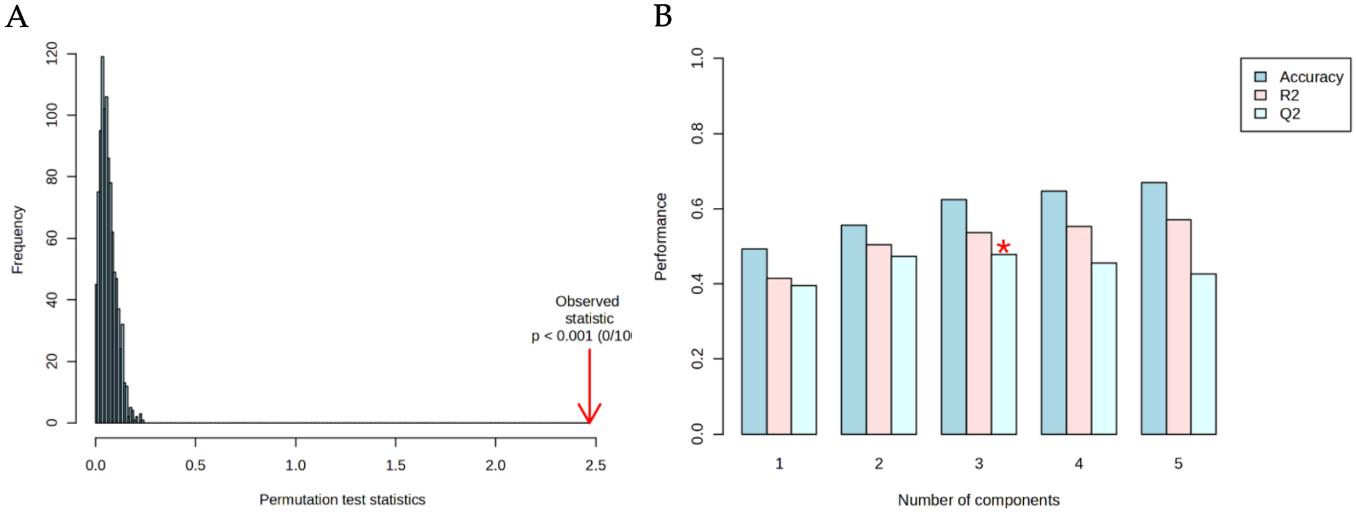

Supplementary Figure S1. Validation of the Partial Least Squares- Discriminant Analysis (PLS-DA) Model. A) A permutation test was performed (1,000 permutations) to validate the PLS-DA model. The histogram shows the distribution of the model's performance statistic (Q^2) from models built on randomly shuffled and permuted data. The "Observed statistic" (red arrow) represents the Q^2 from the model built with the correct, non-permuted data. The observed statistic falls far outside the distribution of the permuted results, indicating the model is statistically significant ($p < 0.001$) and not a result of random chance. B) A 5-fold cross-validation was performed to determine the optimal number of components for the model. The plot displays three performance metrics: Accuracy, R^2 (goodness-of-fit), and Q^2 (predictive ability). The Q^2 value, which represents the model's predictive power, reaches its maximum value with 3 components. This indicates that a 3-component model is the most robust and predictive for this dataset, avoiding the risk of overfitting seen with a higher number of components.

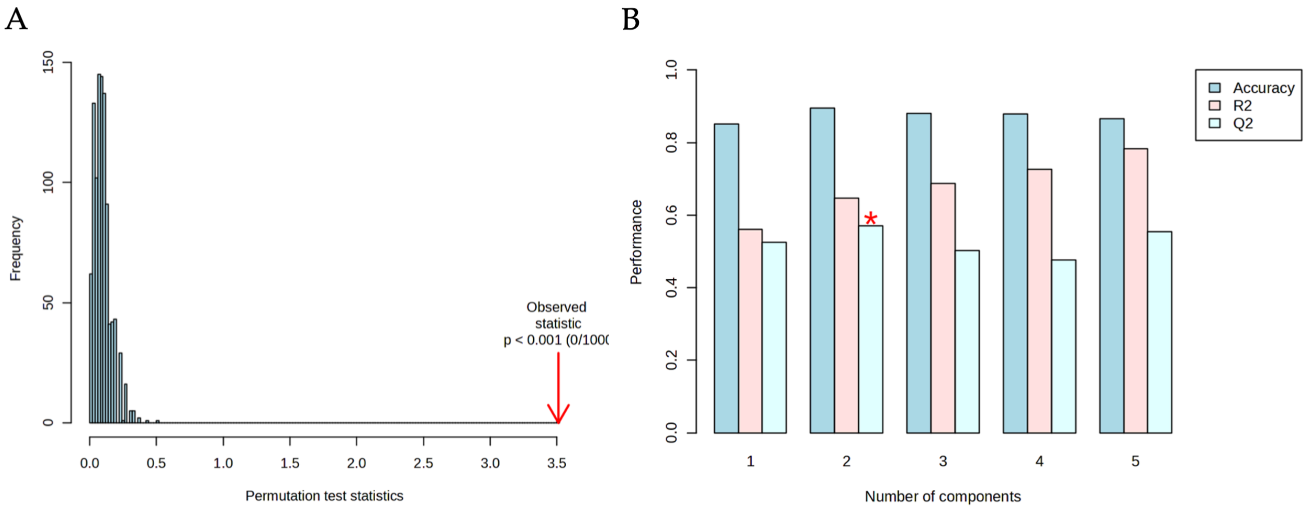

Supplementary Figure S2. Validation of the PLS-DA Model for Pre-Surgery versus Post-Surgery Samples. A) A permutation test (1,000 permutations) was performed to validate the PLS-DA model comparing Pre-Surgery and Post-Surgery samples. The histogram displays the performance distribution of models built on randomly shuffled data, while the "Observed statistic" (red arrow) represents the performance of the actual model. The result ($p < 0.001$) confirms that the separation between the two groups is highly significant. B) 5-fold cross-validation was used to determine the optimal number of components. The plot shows that the model's predictive ability (Q^2) peaks with 2 components. This indicates that a 2-component model is the most robust and predictive for comparing the Pre- and Post-Surgery lipidomic profiles.

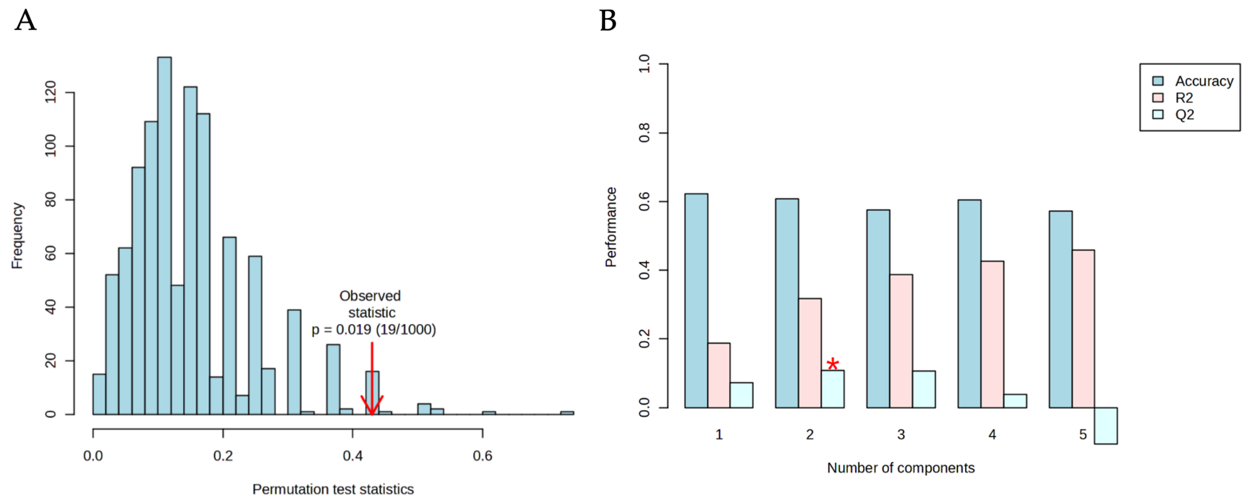

Supplementary Figure S3. Validation of the PLS-DA Model for Pre-Radiation versus Post-Radiation Samples. A) A permutation test (1,000 permutations) was performed to validate the PLS-DA model comparing Pre-Radiation and Post-Radiation samples. The test shows that the separation between the groups is statistically significant ($p = 0.019$), as the observed statistic falls outside the bulk of the distribution from randomly shuffled data. B) 5-fold cross-validation was used to determine the optimal number of components. The model's predictive ability (Q^2), a measure of its robustness, is low for all component numbers and peaks at a value of approximately 0.1 for the 2-component model. A negative Q^2 value for the 5-component model suggests overfitting. Overall, the low Q^2 values indicate that while some statistical separation exists between the groups, the model has poor predictive power.
